# Trajectory-aware risk stratification of oral lichen planus using a multimodal large language model: a longitudinal diagnostic accuracy study

**DOI:** 10.1038/s41598-026-65667-2

**Published:** 2026-08-13

**Authors:** Fatma E. A. Hassanein, Salma M. Saad, Radwa R. Hussein, Asmaa Abou-Bakr

**Affiliations:** 1 Oral Medicine, Periodontology, and Oral Diagnosis, Faculty of Dentistry, King Salman International University, El-Tor, Egypt; 2 Oral Medicine and Periodontology Department, Faculty of Dentistry, Cairo University, Giza, Egypt; 3 Oral Medicine and Periodontology, Ain Shams University in Egypt, Cairo, Egypt; 4 Oral Medicine and Periodontology, Faculty of Dentistry, Galala University, Suez, Egypt

**Keywords:** Oral lichen planus, Artificial intelligence, Large language model, Risk stratification, Longitudinal monitoring, Cancer, Diseases, Medical research, Oncology

## Abstract

To evaluate the performance of a multimodal large language model (LLM) for longitudinal trajectory classification and risk stratification of oral lichen planus (OLP), compared with expert panel consensus. This retrospective diagnostic accuracy study included 300 patients with histopathologically confirmed OLP and at least 24 months of follow-up. Multimodal longitudinal case profiles (serial clinical records, intraoral photographs, and histopathology reports) were independently assessed by (ChatGPT, OpenAI) and an expert panel (the reference standard), both blinded to the results. The primary outcome was sensitivity for the detecting expert-defined high-risk cases (one-versus-rest). Secondary outcomes included overall trajectory classification and three-level risk stratification. Expert consensus classified 156 cases (52.0%) as stable benign, 92 (30.7%) as inflammatory progression, and 52 (17.3%) as suspicious malignant evolution. Risk stratification was 73 low (24.3%), 161 moderate (53.7%), and 66 high (22.0%). For high-risk detection, sensitivity was 78.8% (95% CI 67.2–87.5), and specificity was 99.6% (95% CI 97.6–100.0). Overall trajectory classification accuracy was 94.7%. Three-level risk stratification accuracy was 76.3%, with most errors representing downward shifts (98.6%). The multimodal LLM showed high concordance with expert consensus for longitudinal OLP surveillance, with high accuracy of trajectory classification and high specificity for high-risk identification. These findings suggest that multimodal LLMs may support trajectory-based assessment through integration of longitudinal clinical information, although prospective external validation remains necessary.

**Supplementary Information:**

The online version contains supplementary material available at 10.1038/s41598-026-65667-2.

## Introduction

Oral Lichen Planus (OLP) is a chronic immunological disease with a varied clinical course, ranging from benign to malignant transformations^1,2^. Current OLP surveillance is largely dependent on serial clinical observations, with disease trajectory assessments determined by subjective clinical judgment of morphological changes^3,4^. Of note, these clinical observations are subject to variability, bias, and delays, specifically when performed outside specialty environments.

Multimodal Large Language Models (LLMs) can analyze text and photographs, representing a paradigm shift in chronic disease management^5–7^. While pilot studies have validated the accuracy of multimodal LLMs for single-time-point classification of oral lichenoid conditions^8,9^, their use in longitudinal surveillance remains unexplored.

Our recent study has validated the diagnostic accuracy of multimodal LLMs for tripartite differentiation of OLP, oral lichenoid lesions (OLL), and malignant transformation at a single time point^8^. However, the most important question of whether these models can perform longitudinal risk stratification and disease trajectory classification over time remains unanswered. The possibility of successively scrutinizing, comparing, and analyzing clinical documentation, patient self-assessment, and follow-up photos may aid in objectively assessing the disease activity.

The chronic and progressive nature of OLP requires adequate and standardized follow-up management to ensure maximal benefits to patients. However, monitoring of this condition is limited by inter-rater and intra-rater variability in clinical examinations and the lack of standardized quantitative monitoring scales^9^.

Previous studies have evaluated both LLMs and convolutional neural network (CNN) techniques for diagnosing OLP against OLL or squamous cell carcinoma (SCC) at a single instance^10–12^. To date, there has been no attempt to analyze these techniques for monitoring progression, evolution, and regression across serial visits. There are no studies examining the capability of artificial intelligence (AI) solutions in combining baseline and follow-up information for disease trajectory classification and risk stratification.

The current AI technology in oral medicine tends to use isolated data types, for example, images or text alone. The ability of multimodal LLMs to incorporate information from serial images, longitudinal clinical notes, and the patient’s history for longitudinal disease monitoring remains largely underexplored^13,14^. This research aimed to fill these gaps by validating a multimodal LLM platform for the longitudinal surveillance of OLP. In contrast to previous research efforts that focused on the diagnosis of a single point in time, we aimed to assess the model’s ability to integrate longitudinal clinical information, such as progress notes, interval photos, and histopathology, to dynamically classify trajectories and stratify malignant transformation risk, addressing the critical need for a standardized and objective tool to assist clinicians in the longitudinal follow-up of OLP.

Unlike previous studies that focused on single-time-point classification, we evaluated a multimodal AI framework that incorporates longitudinal clinical information for risk stratification and trajectory classification. This is a step towards moving beyond static diagnostic paradigms and towards longitudinal multimodal assessment.

## Materials and methods

### Study design

This was a retrospective longitudinal diagnostic accuracy study designed to evaluate the performance of a multimodal large language model (LLM) in longitudinal risk stratification and trajectory assessment of oral lichen planus (OLP). The study assessed concordance between LLM-based classification and expert panel consensus using serial clinical documentation and follow-up data. Reporting adhered to the STARD-AI (Standards for Reporting Diagnostic Accuracy Studies for Artificial Intelligence) guidelines. The pre-specified primary endpoint was sensitivity for the detection of expert-defined high-risk cases (one-versus-rest). Secondary endpoints included overall trajectory classification accuracy and three-level risk stratification performance.

### Study setting

This retrospective diagnostic accuracy study was conducted using archived clinical records from three academic institutions: the Faculty of Dentistry, King Salman International University (El-Tor, Egypt), the Faculty of Dentistry, Galala University (Suez, Egypt), and the Faculty of Dentistry, Ain Shams University (Cairo, Egypt). Eligible cases were identified through a systematic review of electronic health records and institutional clinical archives at these centers. All data were de-identified before extraction and analysis. Case selection was performed consecutively according to predefined inclusion and exclusion criteria.

### Ethics approval and consent to participate

The study protocol was reviewed and approved by the Institutional Review Board of King Salman International University (approval number: IRB014-2025). The requirement for informed consent was waived by the same Institutional Review Board due to the retrospective design and use of fully de-identified archival clinical data, in line with applicable national regulations governing retrospective research. The study was conducted in accordance with the Declaration of Helsinki.

#### Informed consent for expert participation

All the experts participating in the reference standard assessment process were well informed regarding the objectives and the concept of the study. The experts participated in the study voluntarily and gave their consent to contribute their evaluations for the research and publication. There is no disclosure of personal details related to the experts in this manuscript.

#### Participants and case selection

Cases were retrospectively identified from institutional electronic health records and clinical archives at the participating centers. Consecutive cases meeting predefined eligibility criteria were screened. Participating centers followed comparable clinical documentation protocols, including standardized intraoral photography and structured clinical records, enabling consistent longitudinal case file construction.

#### Inclusion criteria

Participants were eligible if they:

1. Were aged ≥ 18 years.
2. Had a confirmed diagnosis of OLP based on modified World Health Organization (WHO) clinical and histopathological criteria.
3. Had complete baseline documentation at the index visit (T0), including standardized intraoral photographs, structured clinical notes describing lesion morphology and symptoms, and histopathological confirmation.
4. A minimum follow-up period of 24 months was selected because clinically meaningful trajectory changes and potential malignant transformation in oral lichen planus often occur over extended surveillance periods. Previous longitudinal OLP studies have reported follow-up durations of approximately 4–5 years or longer when evaluating disease progression and malignant transformation^15,16^.

For each eligible case, a structured longitudinal case file was constructed incorporating baseline (T0) documentation and all available follow-up clinical notes, interval intraoral photographs, and histopathology reports where applicable. These longitudinal records were used for both index test evaluation and reference standard determination.

#### Exclusion criteria

Cases were excluded if they:

- Represented oral lichenoid lesions with identifiable reversible etiologic factors.
- Had incomplete baseline or follow-up documentation.
- Were lost to follow-up before 24 months.
- Had concurrent autoimmune mucosal disorders that could confound progression assessment.

### Sample size calculation

The study was powered on the clinically critical endpoint of high-risk progression detection. Pilot data (*n* = 23) demonstrated a sensitivity of 0.75 and a high-risk prevalence of 17.4%. To estimate sensitivity with a predefined 95% confidence interval precision (half-width) of ± 0.12, at least 52 outcome-positive cases were required. Based on the observed prevalence, this corresponded to a minimum total sample size of 287 patients. To ensure adequate representation across prognostic strata and compensate for potential exclusions, recruitment continued until 300 consecutive eligible patients were included.

Cases with missing baseline or follow-up information were excluded (*n* = 25). No imputation of missing data was performed; only studies with complete case files that met the inclusion criteria were used.

### Reference standard: expert panel prognostic stratification

Trajectory and risk classifications were assigned retrospectively based on the complete longitudinal course rather than solely on baseline (T0) features. Each panelist independently reviewed the complete longitudinal clinical record for every case, including baseline documentation (T0), serial follow-up clinical notes, interval intraoral photographs, and histopathology reports when available. Panelists were blinded to the LLM outputs and to each other’s initial assessments. Based on a comprehensive evaluation of disease evolution over the minimum 24-month follow-up period, each expert assigned:

- One trajectory classification (stable benign, inflammatory progression, or suspicious malignant evolution), and.
- One risk category (low, moderate, or high).

The expert panel consisted of three specialists in oral medicine with over 10 years of experience diagnosing and treating oral potentially malignant disorders and oral lichen planus. Before conducting the research, the members of the panel were provided with standardized written definitions of all risk categories and all trajectories. Before independent assessment, panelists also assessed representative example cases to ensure consistent application of the predefined classification criteria. Each panelist independently assigned trajectory and risk classifications. Final reference labels were determined by majority agreement (at least two of three concordant ratings).

The use of majority agreement (two out of three agreements) as the reference standard was predefined because this approach would reduce the influence of inter-individual variation among observers without diminishing the importance of the experts’ subjective evaluations. This method is commonly used when evaluating diagnostic accuracy when there is no specific objective reference standard.

In the rare instances where all three panelists assigned different categories, a structured consensus discussion was conducted to reach a final adjudicated classification. Inter-expert agreement before adjudication was quantified using Fleiss’ kappa.

The inter-expert agreement before adjudication was high (Fleiss κ = 0.79, 95% CI: 0.74–0.84). A complete lack of agreement was seen in 11 cases (3.7%), where a structured discussion was used to reach a consensus. Histopathologically documented cases of dysplasia were seen in 41 of the 66 high-risk cases (62.1%), while the remainder were classified based on suspicious features that warranted biopsy (non-healing ulcers, progressive induration, or exophytic lesions).

Trajectory categories were predefined. *Stable benign* indicated no documented morphological progression or dysplastic change during follow-up. *Inflammatory progression* indicated clinical worsening (e.g., expansion of erosive/atrophic areas or increased symptom severity) without dysplasia. *Suspicious malignant evolution* indicated persistent non-healing ulceration, progressive induration, exophytic change, or histopathological dysplasia. Risk levels reflected overall longitudinal behavior: *low* (stable, no dysplasia), *moderate* (persistent inflammatory activity without dysplasia), and *high* (histopathological dysplasia or clinically suspicious progression warranting biopsy).

Incorporation of histopathologic results was made to form just one element of the longitudinal disease chart, and not an entirely separate gold standard at each follow-up point. Repeat biopsies at each surveillance visit cannot be done routinely for OLP because it would neither be ethical nor practical for stable lesions. Furthermore, trajectory group assignment in OLP is based on the overall development over time of the clinical symptoms and signs, along with histopathology. Thus, the final reference group assignments were based on the overall longitudinal trajectory classification.

#### Mitigation of potential information leakage

To minimize the risk of information leakage from histopathological reports, these reports were preprocessed before the longitudinal case files were constructed. Specifically, the explicit histopathological information concerning the presence or absence of “dysplasia” and its different grades, such as “dysplastic,” “mild,” “moderate,” or “severe dysplasia,” was removed from the text. No changes were made to the clinical reports, intraoral photographs, or the data.

The use of this masking technique was meant to prevent any shortcut learning that would enable a multimodal model to perform well through the identification of isolated keywords used for diagnosis, instead of drawing insights from clinical and imaging data over time^17,18^. The objective was to assess whether the model’s classifications were based on information from serial records rather than isolated diagnostic markers.

The masking method was used only on the input data file for the algorithm. The expert panelists were provided access to the unaltered clinical notes, photographs, and pathology reports in the creation of reference standard classifications.

This preprocessing step was carried out using a standard text processing script by one researcher (F.E.A.H.), and this was verified by another researcher (S.M.S.). The rationale behind this process was to minimize the chances that the predictions made by the model were based on isolated high-signal keywords and that it was forced to rely on combined longitudinal clinical and imaging information.

It is noteworthy that although explicit diagnostic terminology was removed, histopathological information is retained, and that, therefore, implicit information cannot be completely excluded.

#### Exact prompt provided

A single predefined prompt was developed before commencement of the study and was used for all cases without modification. Alternative prompts were not evaluated, and no prompt optimization or iterative prompt engineering was undertaken. This approach was intentionally adopted to avoid performance inflation associated with prompt selection and to provide a standardized and reproducible evaluation framework. Consequently, all cases were assessed using identical instructions and input structures.

“You are an oral medicine specialist. A longitudinal case file will be provided to you for a patient who has a confirmed diagnosis of oral lichen planus. It will contain baseline clinical notes, follow-up notes, and possibly photographs and histopathology reports. Based on this complete longitudinal information provided to you, classify this case as:

1. Trajectory category (select one): stable benign/inflammatory progression / suspicious malignant evolution.
2. Risk category (select one): low/moderate/high.

Give a brief explanation for your classification. Do not enter any extra information outside of this classification and explanation.”

### Index test: multimodal large language model framework

The index test consisted of ChatGPT-5.2 (OpenAI, San Francisco, CA, USA), a multimodal large language model capable of processing both textual and image-based inputs. ChatGPT-5.2 was accessed through the ChatGPT web interface during the study analysis period. No API-level customization, fine-tuning, or system-level prompt modification was performed. All cases were evaluated using a predefined fixed prompt. A single predefined prompt was developed before commencement of model evaluation and remained unchanged throughout the study. No prompt optimization, iterative refinement, prompt selection, or response regeneration procedures were performed. Clinical narratives, serial intraoral photographs, and histopathology reports were presented chronologically within a single conversation to preserve the temporal sequence of disease evolution. Because the model was accessed through the web interface rather than an application programming interface (API), technical parameters such as temperature settings, seed values, and backend model configurations were not user-configurable.

#### Case file preparation

For each included patient, a standardized digital case file was constructed incorporating:

- Baseline clinical documentation (T0), including structured clinical narrative and intraoral photographs.
- Serial follow-up clinical notes documenting symptom evolution and morphological changes.
- Follow-up intraoral photographs.
- Histopathology reports when applicable.

All data were de-identified before submission to the model.

#### Model implementation

The longitudinal case file was submitted to the LLM using a predefined fixed prompt. Clinical narratives, serial intraoral photographs, and histopathology reports were presented chronologically within a single conversation to preserve the temporal sequence of disease evolution. The same prompt structure and data presentation format were used for all cases.

#### Longitudinal data structuring and trajectory assessment

For each patient, all available clinical information was organized chronologically from baseline (T0) to the most recent follow-up visit. The longitudinal case file included baseline clinical findings, serial clinical notes, intraoral photographs obtained during follow-up visits, and histopathology reports when available. Information from each visit was presented sequentially to preserve the temporal order of disease evolution. No explicit weighting of individual timepoints was applied. All available visits were presented to the model in chronological order using the same standardized format. Consequently, trajectory assessment relied on the model’s interpretation of patterns across the complete longitudinal record rather than on investigator-defined weighting schemes. Progression was defined as a sustained increase in clinical severity, lesion extent, symptom burden, development of concerning clinical features, worsening histopathological findings, or movement toward malignant transformation risk during follow-up. In contrast, fluctuation referred to temporary changes in disease activity followed by stabilization or return toward the patient’s previous clinical status. The model was instructed to evaluate the overall longitudinal pattern across visits rather than isolated findings at individual timepoints.

#### Repeatability assessment

For assessing the stability of model output in a deterministic environment, each case was run three times independently. These runs were conducted on separate days to account for possible backend variability. For each of these runs, the main classification for each case in terms of trajectory and risk category was noted. Agreement for all three runs was calculated by using Fleiss’ kappa and percent agreement. For each of those runs, in case of disagreement, the majority classification was used. Cases that had three different outputs, if any, were noted separately.

#### Assessment of longitudinal information utilization and textual leakage

To evaluate whether model classifications reflected utilization of longitudinal information rather than reliance on isolated diagnostic terminology, a randomly selected subset of 50 cases (16.7% of the total dataset) was analyzed. For each case, the model-generated explanation was independently reviewed by two investigators (F.E.A.H. and S.M.S.) using a predefined binary scoring framework. Explanations were classified as demonstrating longitudinal information utilization if they contained explicit references to temporal changes between visits, disease evolution, progression, regression, or comparisons between baseline and follow-up findings. Explanations lacking such temporal references were classified as not demonstrating longitudinal information utilization. Inter-rater agreement was assessed using Cohen’s kappa statistic. Disagreements were resolved through discussion with a third investigator (A.A.B.).

The cases were considered to demonstrate use of longitudinal information if the rationale made specific reference to time changes, such as “enlargement of erosive area compared to previous visit,” rather than just using quotes of histopathology vocabulary. Consensus was reached by a third party, A.A.B., in the event of disagreements. All 50 cases showed the presence of at least one specific time reference, supporting the conclusion that the model utilized longitudinal data rather than relying on individual diagnostic vocabulary. No cases were excluded in this analysis, as the initial evaluation was performed on the entire analysis.

### Statistical analysis

Statistical analyses were performed using IBM SPSS Statistics (Version 27.0.1; IBM Corp., Armonk, NY, USA) and Python. Diagnostic performance for trajectory and risk stratification tasks was evaluated using overall accuracy, sensitivity (recall), specificity, precision (positive predictive value), negative predictive value, and F1-score. Exact (Clopper–Pearson) 95% confidence intervals were calculated for accuracy, sensitivity, and specificity. For the primary endpoint (high-risk detection), a one-versus-rest approach was applied to derive true positive, false negative, false positive, and true negative counts. Agreement between the LLM classifications and expert consensus was assessed using Cohen’s kappa and quadratically weighted kappa for ordinal outcomes. Full confusion matrices were generated, and misclassification patterns, including directionality (under- versus over-classification), were analyzed descriptively. All analyses were two-sided, with a significance threshold of α = 0.05.

## Results

### Sample characteristics

**Fig. 1:**
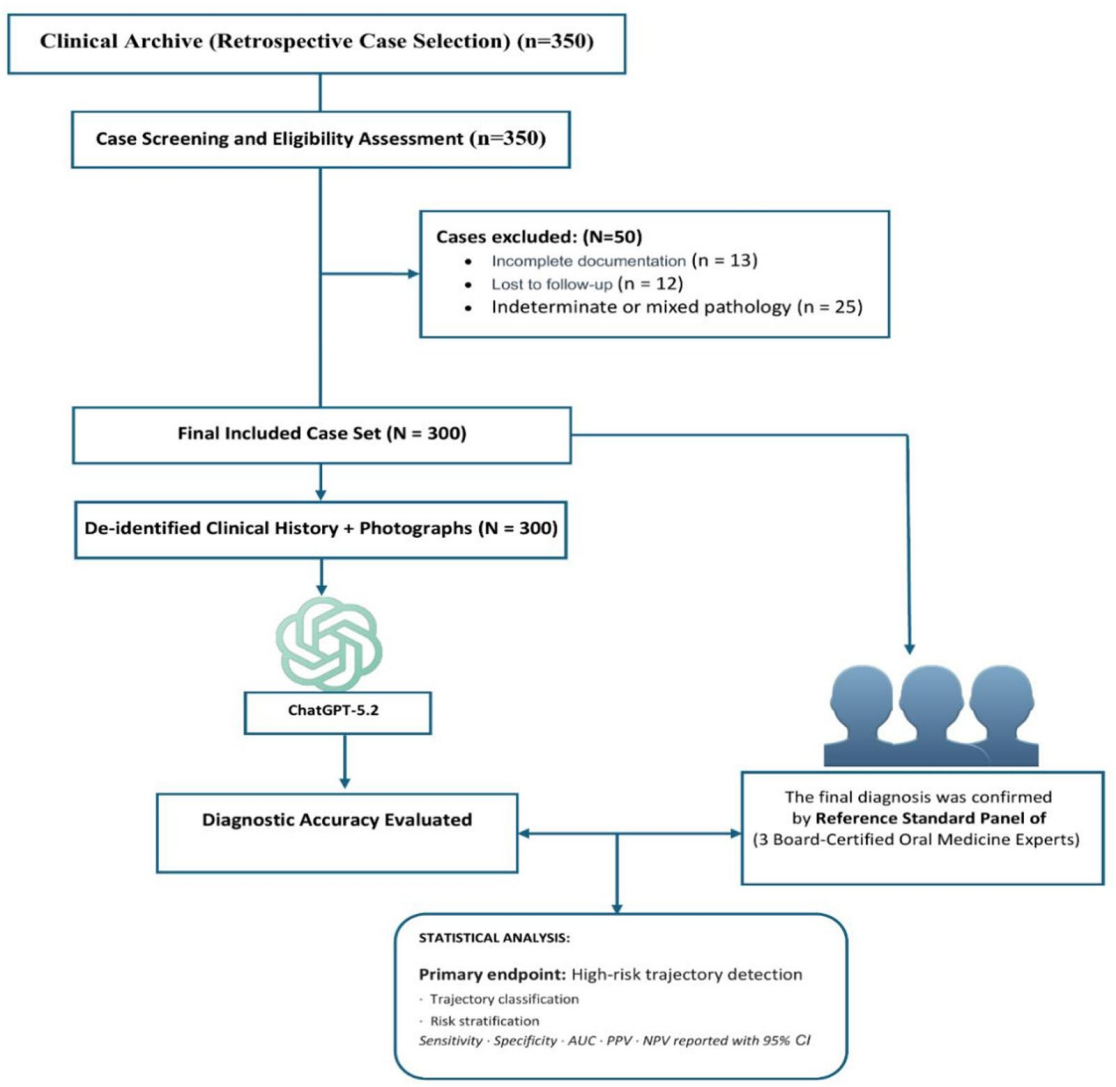
Study flow diagram.

A total of 300 patients with histopathologically confirmed oral lichen planus met the eligibility criteria and were included in the analysis. The study selection process is illustrated in Fig. 1. All patients completed at least 24 months of follow-up. Baseline demographic and clinical characteristics are presented in Table 1. The cohort demonstrated the expected epidemiological profile of oral lichen planus, with a predominance of female patients and representation of multiple clinical subtypes. Patients were recruited from three academic referral centers.

**Table 1:** Baseline demographic and clinical characteristics of the study population (*N* = 300).

Characteristic	Value
Age, mean ± SD (years)	52.8 ± 12.4
Age range (years)	21–81
Female, n (%)	198 (66.0%)
Male, n (%)	102 (34.0%)
Reticular OLP, n (%)	108 (36.0%)
Erosive/ulcerative OLP, n (%)	84 (28.0%)
Atrophic OLP, n (%)	42 (14.0%)
Mixed OLP, n (%)	66 (22.0%)
Center 1, n (%)	124 (41.3%)
Center 2, n (%)	96 (32.0%)
Center 3, n (%)	80 (26.7%)

### Expert reference standard classification

All cases were independently evaluated by ChatGPT (OpenAI) and expert panel consensus, which served as the reference standard. According to expert consensus trajectory classification based on longitudinal follow-up, 156 cases (52.0%) were classified as stable benign trajectory, 92 (30.7%) as inflammatory progression, and 52 (17.3%) as suspicious malignant evolution. Risk stratification classified 73 low-risk cases (24.3%), 161 moderate-risk cases (53.7%), and 66 high-risk cases (22.0%). Expert classifications based on longitudinal follow-up are summarized in Table 2.

**Table 2:** Descriptive comparison of expert consensus and ChatGPT assessments.

Classification category	Subcategory	Expert consensus *n* (%)	ChatGPT *n* (%)	Difference (ChatGPT - Expert)
Trajectory class	Stable benign	156 (52.0%)	143 (47.7%)	-4.3%
	Inflammatory progression	92 (30.7%)	104 (34.7%)	+ 4.3%
	Suspicious malignant evolution	52 (17.3%)	53 (17.7%)	+ 0.4%
Risk level	Low	73 (24.3%)	130 (43.3%)	+ 19.0%
	Moderate	161 (53.7%)	117 (39.0%)	-17.7%
	High	66 (22.0%)	53 (17.7%)	-4.3%

### Primary endpoint: high-risk detection

**Fig. 2:**
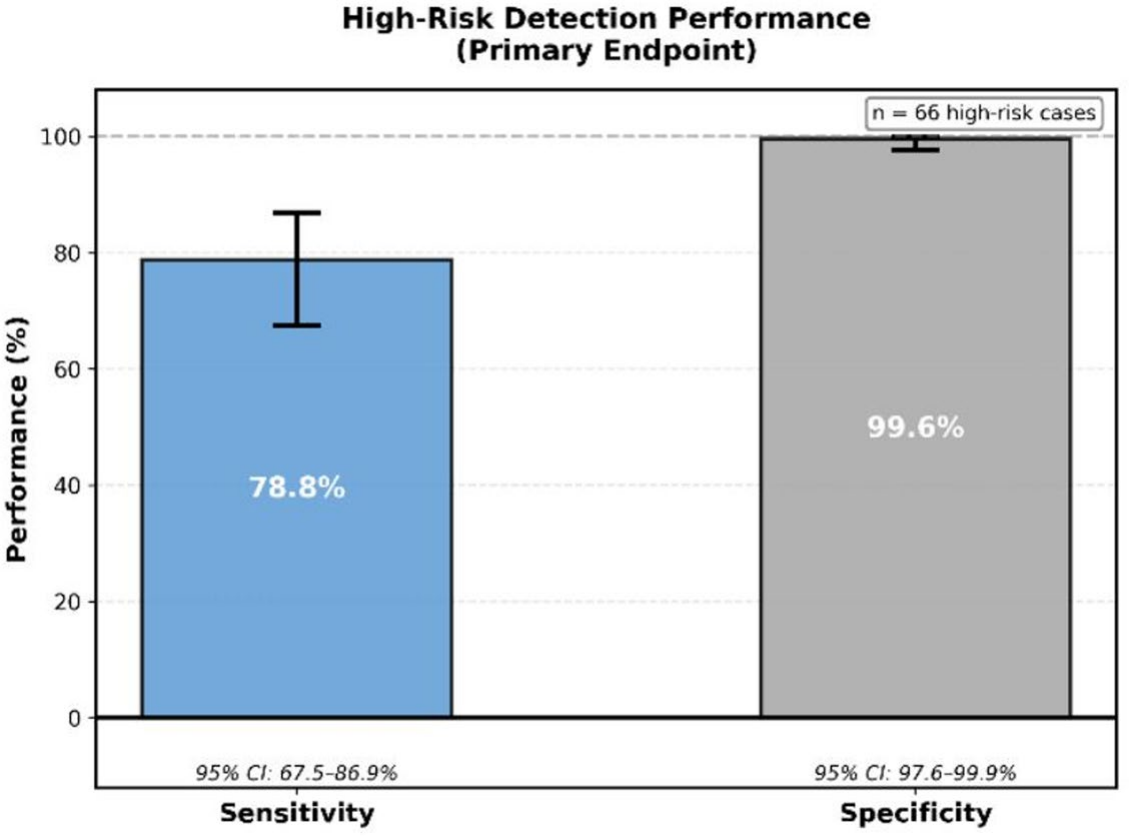
Performance of high-risk detection (primary endpoint).

High-risk detection performance is presented in Table 3 and graphically illustrated in Fig. 2. Among 66 expert-classified high-risk cases, 52 were correctly identified, yielding a sensitivity of 78.8% (95% CI: 67.2–87.5). Fourteen high-risk cases were misclassified (13 assigned to moderate risk and one to low risk). Specificity for high-risk classification was 99.6% (95% CI: 97.6–100.0), corresponding to one false-positive case among non–high-risk cases. The positive predictive value was 98.1%, and the negative predictive value was 94.3%. The one-vs-rest confusion components were TP = 52, FN = 14, FP = 1, and TN = 233.

**Table 3:** High-risk detection performance (primary endpoint).

Metric	Value
Sensitivity	78.8% (52/66) (95% CI: 67.2–87.5)
Specificity	99.6% (233/234) (95% CI: 97.6–100.0)
Positive predictive value	98.1% 98.1% (52/53) (95% CI: 90.1–99.7)
Negative predictive value	94.3% (233/247) (95% CI: 90.7–96.6)

Confusion components (One-vs-Rest): TP = 52, FN = 14, FP = 1, TN = 233.

### Trajectory classification performance

Trajectory classification performance is detailed in Table 4. Overall accuracy for trajectory classification was 94.7% (95% CI: 91.5–96.9). Agreement with expert consensus was high, with Cohen’s κ = 0.913 (95% CI: 0.867–0.955) and weighted κ = 0.936. The macro F1-score was 0.950. Class-specific performance demonstrated a recall of 91.0% for stable benign cases (142/156), 98.9% for inflammatory progression (91/92), and 98.1% for suspicious malignant evolution (51/52). Precision exceeded 87% across all trajectory categories.

**Table 4:** Trajectory classification performance.

A. Overall performance				
Metric	Value			
Accuracy	94.7% (95% CI: 91.5–96.9)			
Cohen’s κ	0.913 (95% CI: 0.867–0.955)			
Weighted κ	0.936			
Macro F1-score	0.950			

B. Class-specific performance				
Class	Precision (%) (95% CI)	Recall (%) (95% CI)	F1-score	Support (n)
Stable benign	99.3 (96.2–100.0)	91.0 (85.4–95.0)	0.950	156
Inflammatory progression	87.5 (79.6–93.2)	98.9 (94.1–100.0)	0.929	92
Suspicious malignant evolution	96.2 (87.0–99.5)	98.1 (89.7–100.0)	0.971	52

Accuracy represents the proportion of correctly classified cases. Cohen’s κ reflects agreement beyond chance. Weighted κ uses quadratic weights to account for the ordinal structure of trajectory categories. The macro F1-score represents the unweighted mean of class-specific F1-scores.

### Risk level stratification performance

**Fig. 3:**
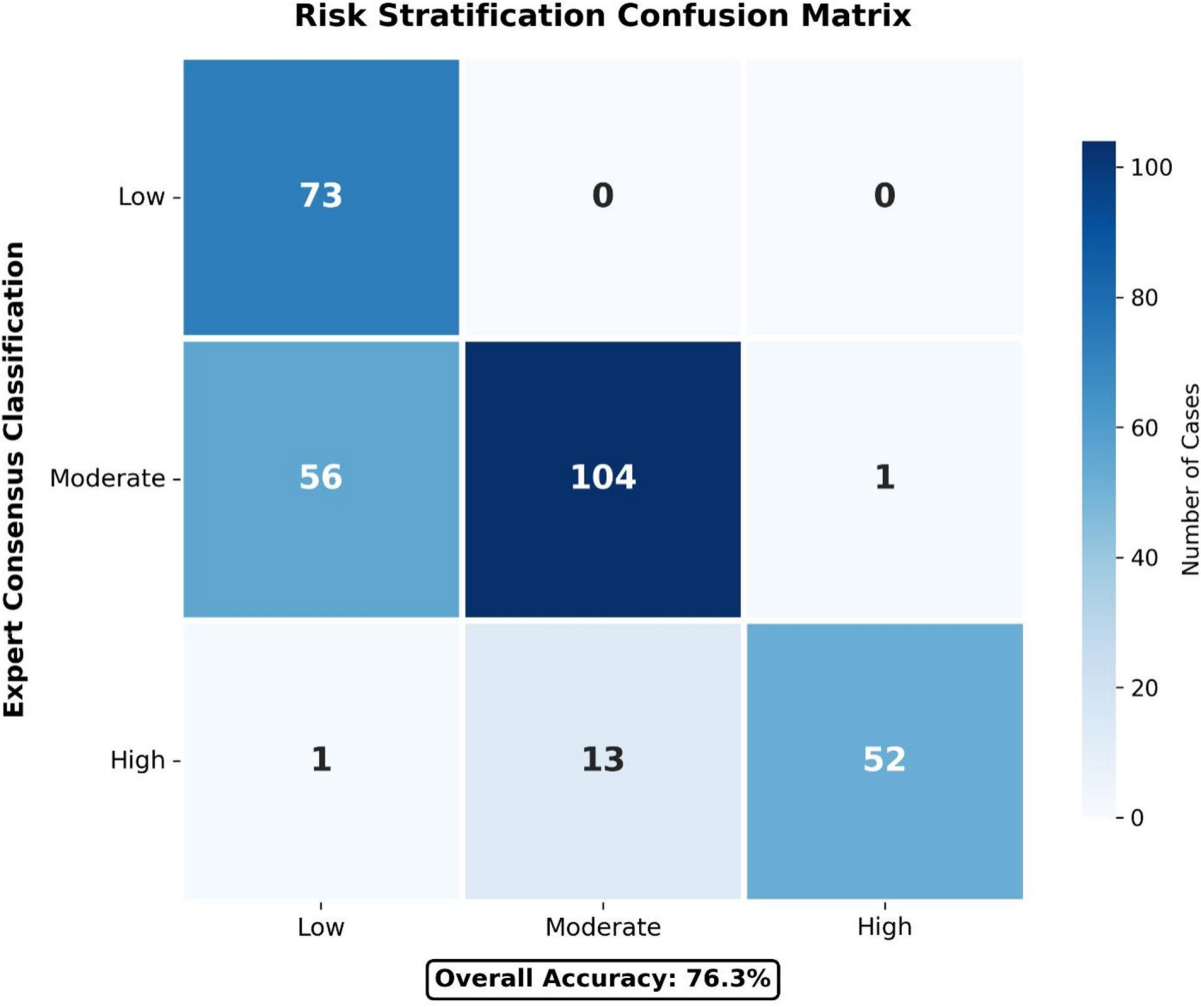
Confusion matrix for three-level risk stratification. Heatmap representation of agreement between expert consensus and the multimodal large language model across low (*n* = 73), moderate (*n* = 161), and high (*n* = 66) risk categories. Most misclassifications occurred between adjacent categories, predominantly moderate-to-low transitions. Overall accuracy = 76.3% (229/300).

Risk stratification performance and the associated confusion matrix are shown in Table 5, with a graphical heatmap representation provided in Fig. 3. Overall, 229 of 300 cases (76.3%) were correctly classified. Class-specific recall was 100.0% for low-risk cases (73/73), 64.6% for moderate-risk cases (104/161), and 78.8% for high-risk cases (52/66). Precision was 56.2% for low risk, 88.9% for moderate risk, and 98.1% for high risk. A total of 71 misclassifications were observed. Most errors represented downward shifts in risk level, including 56 moderate-risk cases assigned to low risk and 13 high-risk cases assigned to moderate risk. One high-risk case was assigned to low risk, and one moderate-risk case was assigned to high risk.

**Table 5:** Risk level stratification performance and confusion matrix.

Expert \ ChatGPT	Low	Moderate	High	Recall (%)	Precision (%)	F1-score	Support (*n*)
Low	73	0	0	100.0	56.2	0.719	73
Moderate	56	104	1	64.6	88.9	0.748	161
High	1	13	52	78.8	98.1	0.874	66

Rows represent expert consensus classification and columns represent ChatGPT classification. Recall corresponds to row-wise sensitivity; precision corresponds to column-wise positive predictive value.

### Error analysis and risk transitions

**Fig. 4:**
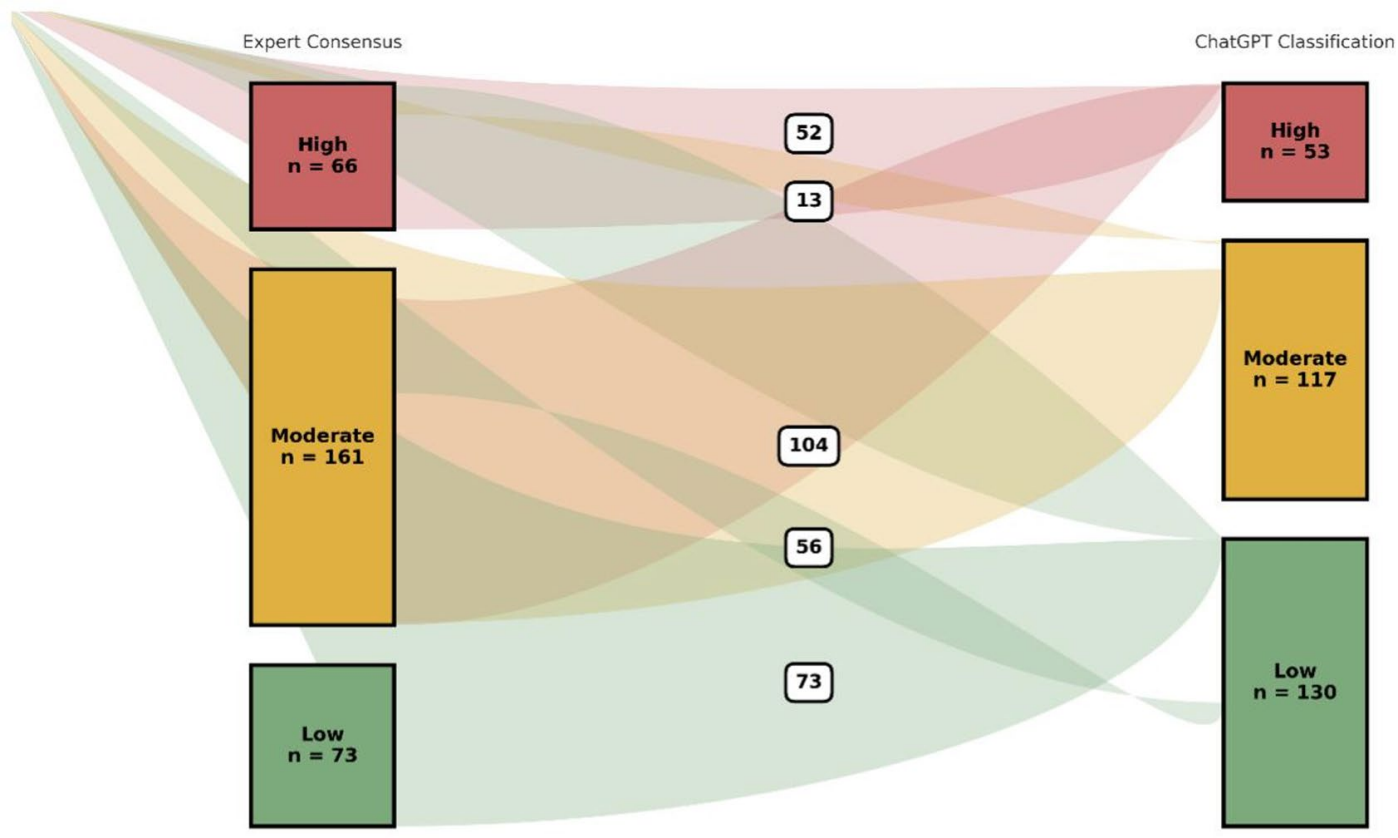
Directionality of misclassification in risk stratification. Distribution of 71 misclassified cases illustrating predominant under-classification (70/71, 98.6%) relative to expert consensus. The most frequent error pattern was moderate-to-low (*n* = 56), followed by high-to-moderate (*n* = 13). One non-adjacent misclassification (high-to-low) and one over-classification (moderate-to-high) were observed.

A total of 71 misclassifications were observed in risk level stratification, corresponding to an overall error rate of 23.7% (71/300). Of these, 70 cases (98.6%) represented under-classification relative to expert consensus, while one case (1.4%) represented over-classification. The most frequent misclassification pattern was Moderate to Low, occurring in 56 cases. Among expert-classified high-risk cases (*n* = 66), 14 were misclassified: 13 were assigned to moderate risk and one was assigned to low risk. A single moderate-risk case was over-classified as high risk. Overall, misclassifications occurred predominantly between adjacent risk categories, with one non-adjacent misclassification (High to Low) observed (Fig. 4). Representative examples of correctly and incorrectly classified cases are provided in Supplementary Table S1 to illustrate typical model successes and failure patterns.

#### Repeatability

Overall, across the three runs, we observed complete agreement among all three runs in 296 out of 300 cases (98.7%), where all three runs agreed on both trajectory and risk classification. Fleiss’ κ measure of inter-rater reliability was 0.96 (95% CI: 0.93–0.99). In the remaining four cases where there were discrepancies between runs, the majority vote was used to reconcile results. No case had three different classifications.

#### Assessment of longitudinal information utilization

Among the 50 randomly selected cases, both reviewers independently evaluated the model-generated explanations using the predefined binary scoring framework. Inter-rater agreement was excellent (Cohen’s κ = 0.89; 95% CI: 0.80–0.98). Following adjudication of discrepant ratings, all 50 cases (100%) were determined to contain at least one explicit reference to temporal changes, disease evolution, or comparisons between visits. These findings provide supporting evidence that the model incorporated longitudinal clinical information when generating classifications rather than relying solely on isolated diagnostic terminology.

#### Calibration analysis for risk stratification

The Brier score was 0.172, indicating a relatively low overall prediction error and generally good agreement between the model’s risk classifications and the expert reference standard. However, the model generated categorical risk classifications (low, moderate, or high risk) rather than continuous probabilistic risk estimates. The Brier score was based on ordinal risk-category encoding and is not to be interpreted as a classical probability-calibration metric. Accordingly, the Brier score should be interpreted as a complementary measure of overall predictive performance rather than a formal assessment of calibration. Consequently, calibration plots and other probability-based calibration analyses were not applicable within the current study framework. For high-risk detection, the area under the receiver operating characteristic (ROC) curve was 0.942 (95% CI: 0.911–0.973), indicating excellent discriminative ability.

## Discussion

This study primarily evaluated the ability of a multimodal large language model to detect high-risk malignant progression in longitudinal OLP surveillance. Our results show that (ChatGPT, OpenAI) provided a high level of agreement with expert consensus for trajectory classification (accuracy 94.7%, κ = 0.913) and high specificity (99.6%) for high-risk malignant progression, although with moderate sensitivity (78.8%) and a marked tendency towards under-classification in risk stratification. These results expand current evidence on AI applications in OLP by addressing the important challenge of longitudinal surveillance^19^.

### Performance of trajectory classification in context

The level of near-perfect agreement between our multimodal LLM and the expert panel for three-level trajectory classification is significantly higher than that reported in previous single-time-point diagnostic studies. Achararit et al. investigated the performance of various CNN models (Xception, ResNet152V2, EfficientNetB3) for binary OLP vs. non-OLP classification on 1,089 clinical images, with accuracies ranging from 81.82% to 88.18%^11^. Similarly, Yu et al. investigated the performance of ChatGPT-5.2, Chat-Diagrams, and Claude Opus for OLP image classification on 128 cases, with overall accuracies of 77%, 80%, and 50%, respectively, following targeted training^20^.

The observed accuracy of 94.7% for three-level trajectory classification suggests a potential benefit of multimodal longitudinal data analysis compared with static image analysis. The observed performance may be due to the incorporation of additional longitudinal data, including clinical, imaging, and histopathologic factors.

These findings are consistent with previous literature reviews stating that multimodal integration of data is a major trend in future AI research related to OLP^19,21^.

### High-risk detection: clinical implications and comparative performance

The main outcome measure of high-risk detection showed a sensitivity of 78.8% and specificity of 99.6%. The high specificity is especially important because false positives could lead to unnecessary biopsies and patient distress^9^. This is different from the performance of recent object detection models. Bharti et al. proposed a YOLOv8 model for the detection of oral potentially malignant disorders and oral cancer from 583 intraoral images, obtaining a recall of 98.24% and a precision of 96.76% for binary classification^22^. Note that their problem was inherently different, as they tackled binary classification based on single-time-point images of clearly localized lesions, in contrast to our model, which tackled three-level trajectory classification based on longitudinal data from OLP patients, including diffuse and bilateral lesions that are harder to analyze computationally^19,22^. Vinayahalingam et al. showed that transformer models (Mask R-CNN with Swin Transformer) had F1-scores of 0.825 and area under the curve (AUC) of 0.948 for the detection of OLP, but again, were limited to a single time-point analysis^23^.

### Under-classification bias and class imbalance

An interesting finding was the tendency toward under-classification, with 98.6% of misclassifications representing downward shifts relative to expert consensus. A likely explanation for this phenomenon is class imbalance, where high-risk instances were in the minority in this dataset, potentially reducing sensitivity to rare but clinically significant outcomes. The observed under-classification bias may reflect model priors or class imbalance effects and warrants further investigation. A false-negative high-risk classification may delay biopsy, enhanced surveillance, or referral.

### Evolution of AI architectures in OLP diagnosis

Our results need to be understood in the context of the evolution of AI architectures for the diagnosis of oral lesions. In early studies, artificial neural networks were used by Jurczyszyn and Kozakiewicz for texture-based differentiation between leukoplakia and lichen planus, and moderate accuracy was achieved through manual feature extraction^24^. Later, CNN-based architectures showed a progressive improvement, and Warin et al. showed that the DenseNet-169 model yielded AUCs of 1.00 for oral squamous cell carcinoma (OSCC) and 0.98 for oral potentially malignant disorders (OPMDs), which was at par with expert performance^25,26^. More recently, transformer architectures such as Vision Transformers and Swin Transformers have shown potential for multiclass classification, and Vinayahalingam et al. showed AUCs of 0.948 for OLP, 0.974 for OSCC, and 0.938 for leukoplakia^23^.

Parola et al. showed that ensemble strategies combining YOLOv8 with Detection Transformer resulted in better performance for neoplastic lesion detection than the individual models^27,28^. Our work fills a distinct niche in this literature, addressing the clinically essential but technically challenging problem of longitudinal risk stratification, which is distinct from previous research focused solely on single-time-point diagnosis and detection^19,21^.

Several limitations of this study should be mentioned. The retrospective nature of this study cannot determine prospective performance in actual clinical practice, where the quality of data may be compromised. The reference standard used the consensus of experts instead of histopathological validation at each point of follow-up, which may introduce some subjectivity.

### Limitations and clinical implications

Several limitations should be considered when interpreting these findings. First, this was a retrospective study based on medical record analysis and may not fully capture the variability and completeness of prospectively collected longitudinal data. Second, only one multimodal large language model and a single prompting strategy were evaluated; therefore, performance may vary across model versions, providers, and prompting approaches. Third, the study population was derived from three academic referral centers within a single country, and no external validation has yet been performed. Consequently, the generalizability of the findings to other populations, healthcare systems, and clinical environments remains uncertain. Additionally, although the use of the ChatGPT web interface reflects real-world clinical implementation, it limited control over certain technical parameters and may affect exact reproducibility because backend model updates and configuration settings are not fully transparent to users.

Another important limitation relates to case selection. Inclusion was restricted to histopathologically confirmed OLP cases with complete longitudinal documentation and a minimum follow-up period of 24 months. This requirement may have introduced selection bias and spectrum bias by enriching the dataset with well-documented cases managed within specialized referral centers. Consequently, the study population may not fully reflect the variability and complexity encountered in routine clinical practice, where incomplete records and irregular follow-up are common. This may have influenced model performance estimates and should be considered when interpreting the findings. Furthermore, oral lichenoid lesions were excluded from the analysis, which may limit the applicability of the findings to broader oral potentially malignant disorder surveillance settings.

A further limitation relates to the masking of dysplasia-related terminology within histopathology reports. This approach was intentionally adopted to minimize information leakage and encourage longitudinal multimodal assessment rather than direct reliance on explicit pathological labels. However, masking may have altered the information available to the model compared with routine clinical practice. Although assessment of longitudinal information utilization was performed using a predefined scoring framework with independent reviewer evaluation and excellent inter-rater agreement, this approach evaluates model-generated outputs rather than the model’s internal reasoning processes. Therefore, conclusions regarding the mechanisms underlying the model’s use of longitudinal information should be interpreted cautiously. A further limitation relates to calibration assessment. Although a Brier score was reported as an overall measure of prediction performance, the model generated categorical risk classifications rather than continuous probabilistic risk estimates. Consequently, formal calibration analyses, such as calibration plots and calibration slope assessment, were not applicable within the current study framework.

The observed tendency toward under-classification represents another important consideration. Most misclassifications involved assignment of cases to lower-risk categories than those determined by expert consensus. While this conservative prediction pattern may reduce unnecessary escalation of surveillance, it may also delay recognition of patients at increased risk of malignant transformation, reinforcing the need for continued clinician oversight.

Despite these limitations, the proposed framework demonstrated high specificity for identifying high-risk patients and may serve as a useful clinical decision-support tool rather than a fully autonomous diagnostic system. The apparent ability to incorporate longitudinal clinical, photographic, and histopathological information offers a potentially valuable approach for standardized surveillance and risk assessment of OLP. Nevertheless, the moderate sensitivity for high-risk case identification and the observed under-classification tendency underscore the importance of maintaining human expertise in clinical decision-making.

Future research should focus on external validation across geographically diverse populations and healthcare settings, evaluation of additional multimodal AI systems, and development of strategies to improve detection of high-risk lesions. Incorporation of additional data modalities, including histopathological images, molecular biomarkers, and genomic information, may further enhance risk stratification performance. Establishing robust methodological, regulatory, and clinical governance frameworks will also be essential for the safe implementation of AI-assisted longitudinal surveillance systems in routine practice.

## Conclusions

This work has shown that a multimodal large language model can provide high concordance with a longitudinal evaluation framework in OLP surveillance, with high accuracy for three-level disease course prediction and near-perfect specificity for high-risk malignant progression. The observed under-classification bias suggests that current models need to be improved before autonomous use. As AI moves from research to practice, it will be crucial to have close collaboration between clinicians, data scientists, and regulatory authorities to enable early and accurate diagnoses and individualized management for patients with OLP.

## Supplementary Information

Below is the link to the electronic supplementary material.

Supplementary Material 1

## Data Availability

Research data supporting this publication is available from the corresponding author upon request.
