# Supplementary material for: Trajectory-aware risk stratification of oral lichen planus using a multimodal large language model: a longitudinal diagnostic accuracy study
Source: Sci Rep. 2026 Aug 13;16:25294. doi: 10.1038/s41598-026-65667-2 (PMC13473131; doi:10.1038/s41598-026-65667-2)
Supplement: Supplementary file 1 [file 41598_2026_65667_MOESM1_ESM.docx]

**Supplementary Table S1. Representative Examples of Correctly and Incorrectly Classified Cases**

| No. | Key Longitudinal Findings | Expert Classification | ChatGPT Classification | Outcome |
| --- | --- | --- | --- | --- |
| 1 | Reticular OLP remained clinically stable throughout follow-up without dysplasia or suspicious changes. | Stable benign / Low risk | Stable benign / Low risk | Correct |
| 2 | Progressive ulcerative changes with persistent suspicious features and histopathological evidence supporting malignant evolution. | Suspicious malignant evolution / High risk | Suspicious malignant evolution / High risk | Correct |
| 3 | Persistent inflammatory activity without progression to dysplasia; moderate surveillance risk assigned by experts. | Stable benign / Moderate risk | Stable benign / Low risk | Under-classified |
| 4 | Progressive clinical deterioration with features considered high risk by experts. | Inflammatory progression / High risk | Inflammatory progression / Moderate risk | Under-classified |
